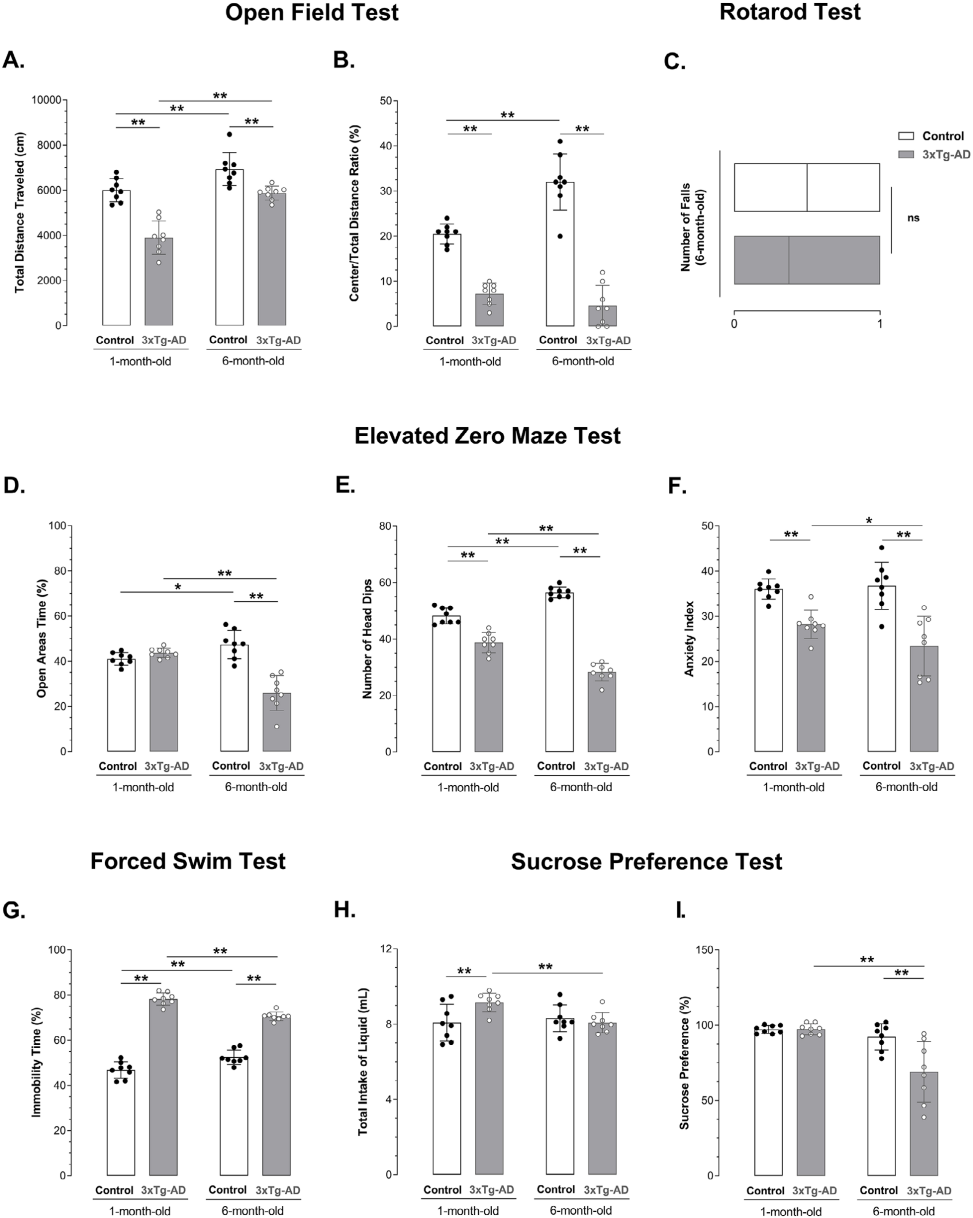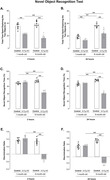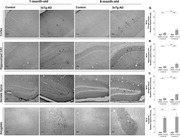# ‘Latent’ mild behavioral impairment as a potential diagnostic strategy for preclinical Alzheimer’s disease: an animal model study

**DOI:** 10.1002/alz.087517

**Published:** 2025-01-03

**Authors:** Orestes Vicente Forlenza, Monique Patricio Singulani, Leda Leme Talib, Rosana Camarini, Luiz Roberto Britto

**Affiliations:** ^1^ Laboratory of Neuroscience (LIM27), Departamento e Instituto de Psiquiatria, Hospital das Clínicas, Faculdade de Medicina da Universidade de São Paulo, São Paulo, São Paulo Brazil; ^2^ Laboratory of Neurosciences (LIM27), Departamento e Instituto de Psiquiatria, Hospital das Clínicas, Faculdade de Medicina da Universidade de São Paulo, São Paulo, São Paulo Brazil; ^3^ Laboratory of Neurosciences (LIM27), Departamento e Instituto de Psiquiatria, Hospital das Clínicas, Faculdade de Medicina da Universidade de São Paulo, São Paulo Brazil; ^4^ Laboratory of Neurochemistry and Behavioral Pharmacology, Departamento de Farmacologia, Instituto de Ciências Biomédicas, Universidade de São Paulo, São Paulo, São Paulo Brazil; ^5^ Laboratory of Cellular Neurobiology, Departamento de Fisiologia e Biofísica, Instituto de Ciências Biomédicas, Universidade de São Paulo, São Paulo, São Paulo Brazil

## Abstract

**Background:**

In Alzheimer’s disease (AD) the fact that neuropsychiatric symptoms can predate the onset of cognitive symptoms suggests that greater focus on the non‐cognitive behavioral changes in earlier life could be an opportunity to investigate ‘latent’ mild behavioral impairment (MBI) as a possible diagnostic strategy for preclinical AD.

**Method:**

We used 1‐ and 6‐month‐old 3xTg‐AD male mice and age‐matched wild‐type animals (CEUA‐ICB/USP: 127/2015). Two batteries of behavioral tests were performed: (1) open field test (OFT), novel object recognition test (NORT), and rotarod test; (2) elevated zero maze test (EZMT), forced swim test (FST), and sucrose preference test (SPT). The immunohistochemistry method was performed to analyze amyloid‐β (Aβ) accumulation (6E10) in areas of the brain. Statistical analysis was performed through two‐way ANOVA followed by a Bonferroni post‐hoc test and unpaired t‐test (IBM SPSS Statistics® software 23). We considered statistically significant p<0.05.

**Result:**

In both OFT (F_(3, 28)_ = 22.92, p<0.001) and EZMT (F_(3, 28)_ = 41.72, p<0.001), a rise in anxiety‐related responses was detected in 3xTg‐AD versus Controls mice from an early age. We also detected depressive‐like behavior in 1‐ and 6‐month‐old 3xTg‐AD evaluated by FST (F_(3, 28)_ = 40.88, p<0.001). In SPT, 6‐month‐old 3xTg‐AD presented a decrease in sucrose preference versus Controls of the same age and versus 1‐month‐old 3xTg‐AD (F_(3, 28)_ = 8.77, p = 0.006), suggesting signs of anhedonia at a later age. NORT was applied to evaluate cognition, which showed 6‐month‐old 3xTg‐AD mice were not able to discriminate the novel from the familiar object in short‐ (F_(3, 28)_ = 132.14, p<0.001) and long‐term memory (F_(3, 28)_ = 275.86, p<0.001) versus Controls. The immunoreactivity analysis showed that 6‐month‐old 3xTg‐AD exhibited a significant increase in Aβ versus 1‐month‐old 3xTg‐AD (parietal cortex, p<0.001; hippocampal CA1, p<0.001; dentate gyrus, p<0.001), justifying occurrence of memory impairment at a late age. When analyzing the amygdala, 1‐ and 6‐month‐old 3xTg‐AD showed similar Aβ immunoreactivity (p = 0.2), suggesting that premature accumulation of Aβ in amygdala can be contributed to dysfunction in emotional responses.

**Conclusion:**

Taken together, we propose that anxiety‐like and depressive‐like behaviors can be detected at an early age in 3xTg‐AD mice as a ‘latent’ MBI syndrome, positioning it as a possible diagnostic strategy for preclinical AD.